# Unusual Response of Thin LiTaO_3_ Films to Intense Microwave Pulses

**DOI:** 10.3390/ma12213588

**Published:** 2019-10-31

**Authors:** Haojia Chen, Qiong Gao, Baoliang Qian, Lishan Zhao

**Affiliations:** 1 College of Advanced Interdisciplinary Studies, National University of Defense Technology, Changsha 410073, China; 15243644199@163.com (H.C.); blqian@163.com (B.Q.); 2 Luoyang Electronic Experiment Testing Centre, Luoyang 471003, China; gaoqiong1980@126.com

**Keywords:** dielectric, pyroelectric effect, ferroelectric materials, LiTaO_3_, polarisation

## Abstract

Fundamentally different responses of a LiTaO${}_{3}$ thin film detector are observed when it is subjected to short microwave pulses as the pulse intensity is altered over a wide range. We start from weak microwave pulses which lead to only trivial pyroelectric peak response. However, when the microwave pulses become intense, the normally expected pyroelectric signal seems to be suppressed and the sign of the voltage signal can even be completely changed. Analysis indicates that while the traditional pyroelectric model, which is a linear model and works fine for our data in the small regime, it does not work anymore in the large signal regime. Since the small-signal model is the key foundation of electromagnetic-wave sensors based on pyroelectric effects, such as pyroelectric infrared detecters, the observation in this work suggests that one should be cautious when using these devices in intense fields. In addition, the evolution of detector signal with respect to excitation strength suggests that the main polarisation process is changed in the large signal regime. This is of fundamental importance to the understanding on how crystalline solids interact with intense microwaves. Possible causes of the nonlinear behaviour is discussed.

## 1. Introduction

How dielectric materials respond to electromagnetic waves has long been a key topic in the physic research field. To date, a relatively complete theoretical framework was established on how they respond to weak and intense lasers, weak microwaves and static or slowly varying electric fields [1]; however, much less is known about how they respond to intense microwaves. This is partially because, traditionally, knowing these materials’ small signal response is adequate for most practical applications, such as microwave communications [2,3,4,5], and facilities needed for this type of experiments (e.g., microwave sources of high-power output and a microwave chamber) are complicated and expensive. Another reason is that microwaves have characteristic frequencies even much lower than soft-phonons in crystalline solids, making many researchers believe that they may have a limited ability to cause effects that are not well-known yet. In this work, however, we show that when intense microwave pulses are really shed on a thin film sample of LiTaO${}_{3}$, which is a widely used dielectric and pyroelectric material, it gives dielectric response that is not only new but also theoretically challenging.

LiTaO${}_{3}$ is a material that has wide applications; however, many of its fundamental properties are still not fully characterised and understood. This work focused on the material’s response of polarisation to microwave excitations.To start from a point that is easy to be understood, we begin with weak microwave pulse excitations and increase the peak field magnitude gradually, and the sample’s response suggests that in the small signal regime the traditional theoretical model is adequate to explain the data; however, in the large signal regime it is not the case. The results in the large signal regime is important because it concerns fundamental understanding on the polarization mechanisms in crystalline dielectric materials. As the area of how intense transient electric fields and nonlinear phonons interact with solids attracts increasingly more attention [6,7], this work can likely lead to a series of following work in this direction. Practically, the results in this work is also of importance because they indicate that caution is needed when pyroelectric-effect-based devices are applied in circumstances, such as electromagnetic waves detection, where a large signal input may be encountered.

## 2. Materials and Methods

The material chosen in this work, LiTaO${}_{3}$, is a pyroelectric and a ferroelectric material, which has a wide range of applications in, for example, fire alarms, laser-energy meters, infrared (IF) thermal imaging, radiometers and energy harvesting [8,9,10,11,12], the common principle underlying all of which is the pyroelectric effect, an effect that was explained many decades ago [8,13,14]. The material’s electric-optical properties also enable many of its applications, for example, serving as electric-optic crystals. Despite of its successful applications in a wide range, LiTaO${}_{3}$ is still far from being full understood in terms of its fundamental properties. Regarding polarisation process in external fields, the material itself and similar compounds, such as LiNbO${}_{3}$, are good platforms for studying dielectric phenomena in ferroelectrics [15,16,17].

**Figure 1:**
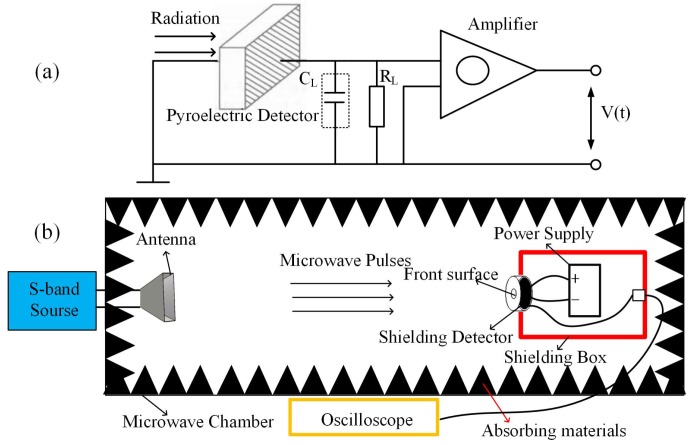
(**a**) signal readout circuit and (**b**) the schematic diagram of the experimental setup. Detailed explanation of it is available in the context.

A thin film sample of LiTaO${}_{3}$ is used in this work, which is made of a poled single crystal whose thickness is 20 μm. Each wide side of it is coated by a thin gold black layer (thickness ≈ 100 nm) as conductive electrode through which the surface charge variation is monitored. The sample is prepared such that its spontaneous polarisation direction is perpendicular to the film surfaces. The incident direction of microwaves is also perpendicular to the film surfaces. As is shown in the lower panel of Figure 1 to readout the polarisation response of the sample to microwave excitations, a resistor with resistance R${}_{L}$ is added in parallel with the sample, and the potential difference across it is monitored and then amplified by a subsequent low noise amplifier (LNA) whose gain *G* = 33.4 dB. The resistor R${}_{L}$ is also accompanied by a parasitic capacitance C${}_{L}$; however, it can be mathematically neglected because its value is much smaller than that of the capacitor formed by the film sample and its conductive coating layers. By integrating these elements together one essentially obtains a film detector, and parameters of the detector is listed in Table 1.

To study the detector’s response to microwave pulses, one needs to make sure that only the functional surface of the LiTaO${}_{3}$ detector can be exposed to incident microwave beams because coupling between the incident beams and the wiring or other parts of the setup can easily result in unwanted signals. To ensure this, the detector is shielded by an aluminium can, which has a circular window leaving the functional surface of the detector to be exposed in incident microwave beams, as is shown in panel (b) of Figure 1. Battery power supply of the detector and necessary wiring also need be properly shielded, thus a larger aluminium box is applied to accommodate them (indicated by the red box in panel (b) of Figure 1). On the back side of the shielding box, a coaxial connector is installed to enable the voltage signal of the detector to be transferred to an oscilloscope through a shielding cable. The detector and the shielding box are placed inside a microwave chamber, and are located in the far filed region of an antenna that is connected to a microwave generator. The position of the antenna and the microwave generator is illustrated in panel (b) of Figure 1. The latter, which works in S-band (2.8 GHz) and can provide as much as 1MW peak power of short microwave pulses with maximum pulse duration of a few microseconds, is the source of intense microwave beams. When microwave beams are shed on the detector, the output of the detector is read and recorded by an oscilloscope that is placed in an independent shielding room next to the microwave chamber.

## 3. Results and Discussion

**Figure 2:**
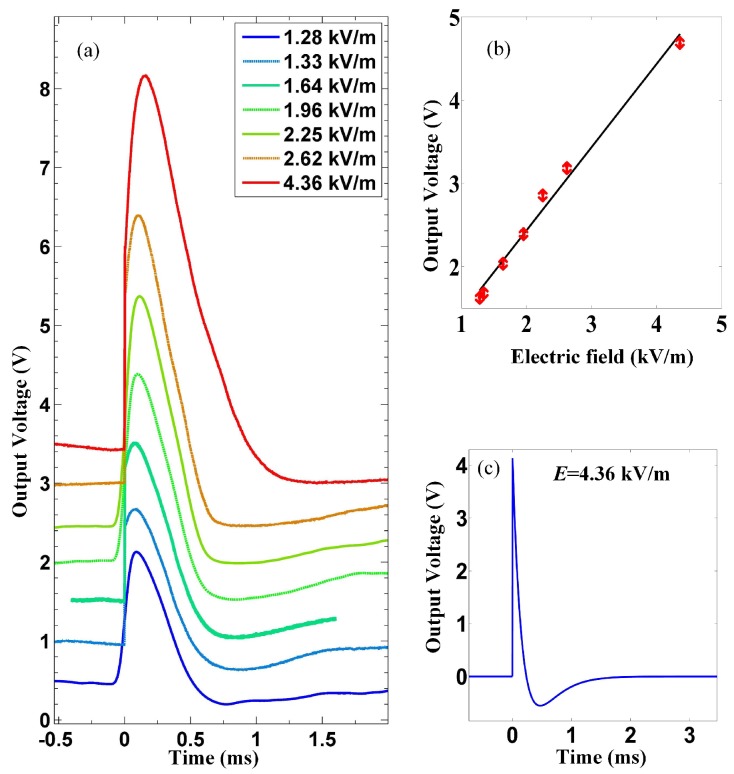
The measured and the calculated voltage response of the LiTaO${}_{3}$ detector to short rectangular microwave responses, whose duration is kept at 2.2 μs while the magnitude varies. In (**a**), the seven curves represent the voltage response at seven different peak field magnitudes below or equal to 4.36 kV/m, respectively. (**b**) shows the linear relationship between the the maximum of output voltage and the peak magnitude of the incident pulses. (**c**) shows the theoretical prediction of the detector’s voltage response to a pulse of 4.36 kV/m (with absorption coefficient η = 12%), whose magnitude agrees with that of the red curve in Figure 2a.

As mentioned in the first section, to start from a point that is easy to be understood, we begin with relatively weak microwave pulses. Figure 2a shows the detector’s output voltages as a function of time *t* when the duration of incident microwave pulses $\tau_{p}$ is maintained at 2.2 μs while their peak magnitude *E*${}_{max}$ varies between 1.33 kV/m and 4.36 kV/m. Figure 2b shows the relationship between the maximum of output voltage *V*${}_{max}$ and *E*${}_{max}$, and it is clearly linear. Such linear relationship indicates constant voltage responsivity of the detector, which is expected. This part of data is trivial since the traditional pyroelectric model is readily adequate to quantitively explain it, as explained below.

To interpret the observed responsivity quantitatively, Equations (1)–(3) need be applied, which together form the mathematical description of a pyroelectric detector [13].

(1) $$\begin{array}{c} C_{th}\frac{dT(t)}{dt}+G_{th}T\left(t\right)=\eta \Phi \left(t\right) \end{array}$$

(2) $$\begin{array}{c} I_{p}\left(t\right)=pA\frac{dT(t)}{dt} \end{array}$$

(3) $$\begin{array}{c} C\frac{dV(t)}{dt}+\frac{V(t)}{R}=I_{p}\left(t\right) \end{array}$$

Equation (1) describes how the radiation flux Φ(t) changes the temperature *T*(*t*) of the detector, where $C_{th}$ and $G_{th}$ represents the thermal capacity and the thermal conductance of the detector respectively, and η is the absorption coefficient. Equation (2) describes the pyroelectric effect in which the temperature variation of the detector causes a net current *I*(*t*) passing through the electrodes. In Equation (2), *p* is the pyroelectric coefficient of LiTaO${}_{3}$ and *A* is the surface area of the LiTaO${}_{3}$ film. Equation (3) describes the relationship between the output voltage *V*(*t*) and the electric current *I*(*t*), where *R*${}_{L}$ represents the resistance of the resistor connected to the LiTaO${}_{3}$ film capacitor and *G* is the gain of the subsequent low noise amplifier.

The radiation flux Φ(t) can be calculated with the measured power of a small antenna in adjacent to the detector and the surface area of the LiTaO${}_{3}$ film. With Φ(*t*) and η known, Equations (1)–(3) can be worked out using the detector parameters listed in Table 1. For a short rectangular-shaped microwave pulse, for example when *E*${}_{max}$ = 4.36 kV/m and $\tau_{p}$ = 2.2 μs, the calculated voltage response, after taking into account the gain of the amplifier, is shown in Figure 2c, assuming η = 12%. It is seen that with the assumed η the magnitude of the calculated voltage response agrees with the measured value which is illustrated by the red curve in Figure 2a. Such low absorption coefficient is not unreasonable for two reasons. One is that for the portion of microwaves penetrated into the thin electrode layer, little is absorbed because the loss tangent of LiTaO${}_{3}$ is less than 0.1% [18]. The second is that the energy absorbed by the electrodes is not much because the wavelength of incident microwaves is higher by several orders than the electrode film thickness and radiation reflection and penetration takes away most of the incident energy (The penetration depth for microwaves is at least higher by an order than the gold black layer thickness which is ≈100 nm). This indicates that the data shown in Figure 2 of the film detector shows no obvious deviation from the traditional model for a pyroelectric detector, and is thus trivial.

**Figure 3:**
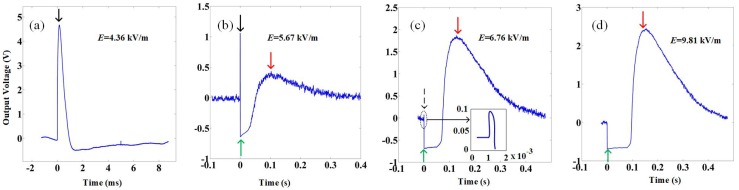
The measured output *V*(*t*) of the LiTaO${}_{3}$ detector at four different values of *E*${}_{max}$, while the pulse duration is kept at 2200 ns. Panel (**a**) repeats the data of *E*${}_{max}$ = 4.36 kV/m shown in Figure 2a. Panel (**b**–**d**) show the data of *E*${}_{max}$ increased from 5.67 kV/m to 9.81 kV/m. The black arrows indicate that as *E*${}_{max}$ is increased from 4.36 kV/m to 9.81 kV/m, the peak signal, which is expected with pyroelectric effects, is suppressed gradually. The green arrows indicate signals in opposite direction of the original peak develop as *E*${}_{max}$ increases, and the red arrows mark the appearance and development of a new hump of *V*(*t*) at large *E*${}_{max}$.

However, when *E*${}_{max}$ is further increased, the output voltage *V*(*t*) does not increase accordingly anymore, and complicated behaviour appears, indicating altered responsivity. Indeed when *E*${}_{max}$ reaches 5.67 kV/m and beyond, *V*(*t*) shows qualitatively changed voltage response, as is shown in Figure 3. Panel (a) of Figure 3 repeats the data at *E*${}_{max}$ = 4.36 kV/m, while panel (b) to panel (d) show data obtained at *E*${}_{max}$ = 5.67, 6.76 and 9.81 kV/m, respectively. As is shown, when *E*${}_{max}$ is increased from 4.36 kV/m to 9.81 kV/m, the original peak signal, which is indicated by the black arrows, is gradually suppressed until it disappears completely (panel (d)). At the same time, signal in opposite direction to the original peak develops gradually, as indicated by the green arrows, and so does a new hump of *V*(*t*) which did not exist at and below *E*${}_{max}$ = 4.36 kV/m, as indicated by the red arrows.

If one starts with a fixed *E*${}_{max}$ and alters $\tau_{p}$, one finds a similar *V*(*t*) evolution process (i.e., enlongation of $\tau_{p}$ results in similar effects as caused by increments of *E*${}_{max}$).

The observed nonlinear voltage response of *V*(*t*) at large *E*${}_{max}$, especially the response marked by the green arrows shown in Figure 3, is unusual and is also the central discovery of this work. The application of microwaves leads to penetration depth that is much longer than the gold black layer thickness and enables direct body interaction between incident beams and the LiTaO${}_{3}$ material itself. It makes local heating of the coating layer due to incident microwave beams of significantly less importance than that in similar studies with lasers, and interesting physics beyond heat transfer process of normal pyroelectric detectors is permitted.

Hints of the negative part of *V*(*t*) were noticed by C. B. Roundy when a LiTaO${}_{3}$ detector was subjected to an intense 1.06 μm laser pulse, but was attributed to damped ringing effects [14]. However, from Figure 3 it is seen that in the large signal regime of microwave pulse excitation, ringing effects are unlikely the cause of the strengthened negative voltage response, and the reason is obvious. However, on the other hand, if the negative response of *V*(*t*) is physically nontrivial, its development and the suppression of the original *V*(*t*) signal suggests that some kind of competing mechanism to the pyroelectric polarisation process develops as *E*${}_{max}$ increases, which is yet to be revealed.

**Figure 4:**
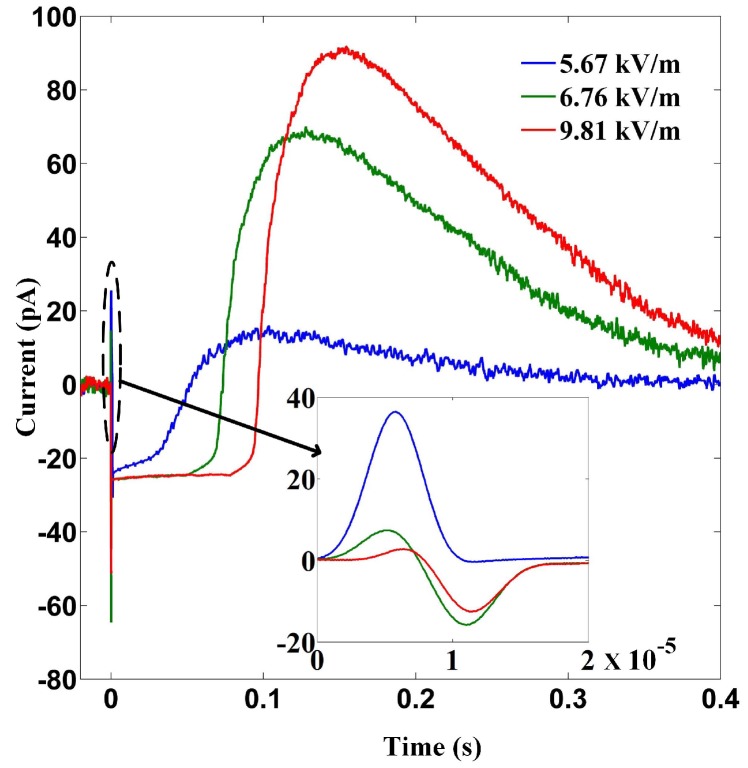
Electric currents between the two film electrodes inferred from the data shown in Figure 3a and Equation (3). The gain of the amplifier is devided when calculating the currents.

**Figure 5:**
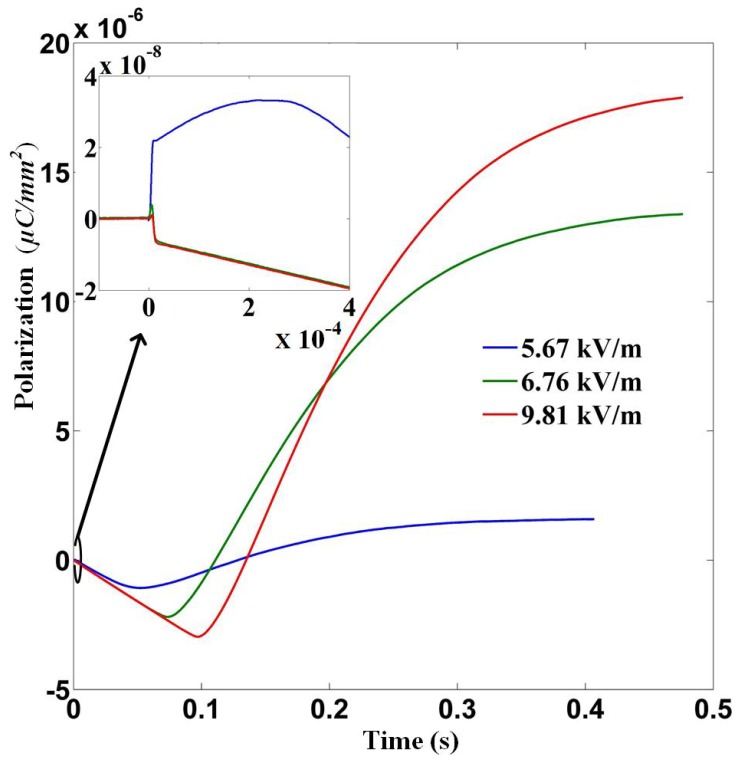
Surface polarisation at a function of time when the sample is excited by microwave fields of three distinct magnitudes. The pulse duration is kept at 2.2 μs.

With Equation (3) and the measured voltage signal, the output current can be calculated. Figure 4 demonstrates the results corresponding to the data shown in Figure 3b–d. In Figure 4 the signal amplification due to the amplifier is removed. Suppression of pyroelectric current and development of current in opposite direction is clearly shown in Figure 4 and emphasised in its inset. The surface polarisation is worked out by integrating the current over time and the result is shown in Figure 5. This, at the first glance, usually means that the polarisation of the LiTaO${}_{3}$ film is not relaxed but strengthened when (and right after) the film is subjected to intense microwave pulses. This is striking because applying an oscillating field (e.g., an electromagnetic field) to a dielectric usually does work to it, leads to temperature increment and relaxes the polarisation to some extent. That is why with the electrocaloric effect [19] the application and removal of an electric field usually leads to positive and negative temperature change respectively. Although there are known examples of exception where applying an electric field (for example a single period of terahertz wave) to a material directly causes temperature lowering [6], that only occurs close to a structural phase transition and the electric field needs be strong enough to drive the material across the phase transition. For LiTaO${}_{3}$, its Curie temperature is about 650 K, which is far above room temperature, at which the temperature the experiment is conducted, and moreover the (static) critical electric field for LiTaO${}_{3}$ is about 41.2 kV/cm [20], which is higher by two orders than the largest *E*${}_{max}$ applied in this work. Therefore there is no obvious reason to expect enhancement of polarisation.

Fundmentally the voltage and current signal of the LiTaO${}_{3}$ film is determined by the surface charge density variation in response to microwave excitations. The surface charge density, in general, is described by *D* = $\epsilon_{0}$*E* + *P*, in which *D* represents the dielectric displacement in unit of C/m${}^{2}$, $\epsilon_{0}$*E* represents the contribution from vacuum where $\epsilon_{0}$ is the vacuum dielectric permittivity, and *P* denotes the material polarisation [21]. Since the vacuum contribution results in no percularity, the material polarisation must account for the unusual behaviour of *V*(*t*) in the large signal regime.

In a thermodynamic approach, the electric displacement corresponds to the electric field differential of the Gibbs free energy *G* at constant stress and constant temperature, and differentiating *D*${}_{i}$ gives [22]

(4) $$dD_{i}={\left(\frac{\partial D_{i}}{\partial T}\right)}_{X,E}dT+{\left(\frac{\partial D_{i}}{\partial X_{jk}}\right)}_{T,E}{\mathrm{dX}}_{\mathrm{ij}}+{\left(\frac{\partial D_{i}}{\partial E_{j}}\right)}_{T,X}{\mathrm{dE}}_{j}\;$$

where X${}_{jk}$ represents the sample stress. where X${}_{jk}$ represents the sample stress. It is a second-rank tensor and X${}_{jk}$ indicates the normal stress while j=k, and the shear stress applied to the vertical plane of the *j*-axis in the direction of the *k*-axis while j≠k. The three terms on the right hand side of Equation (4) corresponds to the pyroelectric, direct piezoelectric and dielectric contribution to the material polarisation, respectively. The pyroelectric effect was discussed above. For a sample that is not clamped, the second term can be neglected. Therefore one is left with the dielectric polarisation when analysing the possible polarisation mechanisms competing with the pyroelectric effect.

Microscopically, there are in general four types of physical mechanisms of polarisation in solids, i.e., electronic polarisation, ionic polarisation, orientation polarisation and space-charge polarisation [1]. Each of them has a characteristic frequency and an energy scale. For example, electronic polarisation occurs at around 10${}^{15}$ Hz, and ionic polarisation, which is related to the molecular or ionic lattices vibrations, usually occurs at frequencies on the order of 10${}^{12}$–10${}^{13}$ Hz. It is noted that the characteristic energy scale of microwaves with frequencies on the order of GHz, is no more than 1 μeV, which much lower than that of even soft phonons, so neither electronic nor ionic polarisation is responsible for the results above. Orientation polarisation occurs in materials with permanent electric dipoles which can be reoriented by external electric fields. For LiTaO${}_{3}$, in the deeply ordered state, the freedom of dipole orientation is limited to its polar axis. Since both the polarisation direction and the microwave incident direction is perpendicular to the film surface, the direction of oscillating electric fields are perpendicular to the polarisation direction because electromagnetic waves are transverse waves. Therefore microwave pulses with incident angle normal to the film surface are unlikely able to substantially change the material polarisation and cause effects that persist even long after the removal of the fields. Space charge polarisation needs be considered carefully.

Domains, defects and trapped charge carriers, which can give rise to space charge polarisation, are commonly seen even in single crystals. Their detailed influence to the polarisation of a specific material often remains a challenging subject to date. The characteristic time of hopping polarisation of, for example, trapped ions (at defects or domain walls), is on the order of 1 μs to 1 s, which is consistent with the time scale of the observed voltage signal, making it a suspicious cause for the nonlinear response in the large signal regime. One possibility is that there may be trapped charge carriers with very shallow energies in the sample that can be excited by the intense microwave fields. If the majority of the generated charge carriers that can diffuse to the electric leads of the sample have the opposite sign of charge to that of those released by the pyroelectric effect, suppression of the pyroelectric current may be expected. The metastable state cannot last long and eventually the sample will come back to its original state, during which process one may observe electric currents with the same direction of the pyroelectric current. This picture is consistent with the observation from Figure 3, Figure 4 and Figure 5. This is also reminiscent of charge transport occurring in LiNbO${}_{3}$ [23], the sister compound of LiTaO${}_{3}$, when it is subjected to laser pulses at UV or visible wavelengths. There, though, the generation of charge carries are reasonably attributed to deep level atomic excitation because the excitation energy is on the order of a few eV [24,25,26].

Regarding domain physics, because of the monodomain nature of the samples used in this work, domain wall dynamics is not expected to play an important role. However, the situation may be complicated if the poling process is incomplete because of, for example, defects, which can change the local electric field environment. In such case there may be microscopic domains in which the electric dipole direction is not perpendicular to the film surface, and the disturbance of microwave fields may leads to polarisation change at these locations or in their neighboring region. It is not clear yet how such process can result in the observed nonlinear response.

If the analysis above is true, it means that the nonlinear polarisation response comes from sources other than intrinsic atomic or lattice excitations, and that implies (A) the threshold peak field strength of the “large signal regime” may vary from sample to sample, although the qualitative trend of polarisation response to microwave pulse strength variation should be similar across different samples, and (B) no obvious frequency preference should be expected in the microwave frequency range, and (C) for a perfect single crystal made of a monodomain, the reported behaviour in this study should not occur. While (C) is difficult to be verified, (A) and (B) turn out to be true for the three samples measured in this work, based on data that will be published in detail later.

It is recognised that the experimental parameter regime in this study belongs to a rarely explored area, where intense time-varying electric fields that can be regarded phononic phonons are applied to a ferroelectric in its deep ordered state. Being far away from a structural phase transition and with the maximum electric magnitude much lower than the coercive force, usually not many interesting dielectric phenomena are expected to occur to a poled ferroelectric material when the excitation frequency is lower than the atomic and lattice excitation frequency by several orders of magnitude. However, it turns out that such kind of studies may bring us information on dielectric processes beyond our naive expectation and it may help us improve our understanding on dielectric phenomena in real materials, which always have imperfections.

## 4. Conclusions

In summary, we reported that for a widely used pyroelectric and ferroelectric material, LiTaO${}_{3}$, when a thin film sample is subjected to intense microwave pulses, unexpected nonlinear polarisation response is observed, while only a linear response is seen when the pulse intensity is low. The linear response in small signal regime can be attributed to pyroelectric effects [27,28,29]. However, the nonlinear response in the large signal regime is more complicated. Analysis indicates that the large signal response are likely due to space polarisation caused by extrinsic sources instead of intrinsic atomic excitations, and trapped charge carriers are potential causes. This work indicates that even when it is far away from a structural phase transition, intense microwave pulses may bring more information of dielectric polarisation than normally expected. Further experimental study and theoretical input is definitely needed to in order to uncover the mechanism responsible for the observed unusual polarisation phenomena.

## Figures and Tables

**Table 1:** Parameters of the LiTaO${}_{3}$ thin film detector.

Name	Value	Unit
pyroelectric coefficient *p*	${}^{1}2.3\times 10^{-4}$	C/(m${}^{2}\cdot$ K)
relative permittivity $\epsilon_{r}$	${}^{1}47$	F/m
specific heat *c*	^1^ 3.2 $\times 10^{6}$	J/(m${}^{3}\cdot$ K)
thermal conductivity *g*	^1^ 4.6	W/(m × K)
active area *A*	$7.85\times 10^{-7}$	m${}^{2}$
film thickness *d*	$20\times 10^{-6}$	m
detector capacitance $C_{d}$	$1.63\times 10^{-11}$	F
detector resistance $R_{L}$	$1.23\times 10^{7}$	Ω

The data of ${}^{1}$ From Ref. [8].
